# Michigan State Board of Veterinary Medical Examiners

**Published:** 1901-11

**Authors:** 


### STATE BOARDS OF VETERINARY MEDICAL EXAMINERS.

### MICHIGAN STATE BOARD OF VETERINARY MEDICAL EXAMINERS.

THE following veterinarians have complied with the rules and regulations of the veterinary law of Michigan, and have registered through the Secretary of the Board in the several counties of that State: J. H. Pear, Saugatuck; W. A. Whitney, Big Rapids; Francis Law, Detroit; A. Elgas, Hertford; A. B. Warner, Portsmouth, Ohio; A. Z. Nichols, Pittsford; W. C. Wooten, Grand Rapids; C. H. Adams, Carson City; W. E. Adams, Carson City; R. W. McDonald, Flint; H. J. Spiers, Jackson; Theodore Lane, Iosco; F. G. Gilbank, Detroit; M. A. Augustin, Chesaning; G. W. Dunphy, Quincy; William Jopling, Owosso; W. A. Griffin, Detroit; J. Hawkins, Detroit; J. J. Joy, Detroit; F. C. Wells, Warren; A. Von Rosenberg, Lansing; Edwin Austin, Romeo; A. B. McCall, Memphis; A. A. Immel, Powers; J. M. Dodge, Elmwood; B. F. Baldwin, Rockford; J. H. Cunnington, Durand; C. D. Rathburn, Sherwood.

